# The Acceptability of Yoga as a Family Intervention: Using Family Journals as a Data Collection Tool

**DOI:** 10.1192/bjo.2024.154

**Published:** 2024-08-01

**Authors:** Hayley Graves, Hannah Merdian, Sharron Smith, Kirsty Miller

**Affiliations:** University of Lincoln, Lincoln, United Kingdom

## Abstract

**Aims:**

The number of children and young people across the UK experiencing mental health difficulties is rising, with 1 in 6 young people aged 6–16 with a diagnosable mental health condition.

The school environment can be a crucial setting for mental health promotion as it can reach such a large number of young people.

The application of mindfulness for wellbeing is becoming increasingly popular. Research demonstrates mindfulness is an effective treatment for many psychological conditions and adding a yoga element is thought to bring additional benefits.

Interventions focusing on wellbeing are found to demonstrate more consistent improvements when parents are involved, supporting the concept of a family intervention.

A 6 week family intervention was developed specifically for children, to promote overall wellbeing.

Aim:

1. To ascertain the acceptability of yoga as a family intervention.
2. To ascertain the acceptability of family journals as method to collect data.

**Methods:**

- 6 weekly, 1hr, yoga and mindfulness intervention.
- 9 children aged 6–11, 9 adults.
- A weekly family journal was used collect data.
- Data from the journals were analysed using Thematic Analysis.
- A Linear Numeric Scale was used pre and post intervention rating 1–5: asking the participants to rate their confidence in relation to breathing techniques, yoga, working as part of a group, and understanding mindfulness.

**Results:**

**1. Acceptability of yoga as a family intervention**

Results from the pre post tests, indicate a significant relationship between the intervention and the participants.

Confidence in yoga P < 0.008 (mean pre test 1, post test 4).

Confidence with breathing P < 0.008 (mean pre test 3, post test 5).

Confidence with mindfulness P < 0.004 (mean pre test 1, post test 4).

Working as part of a group P < 0.004 (mean pre test 3, post test 5).

**2. Acceptability of family journals as a method to collect data**

The journals provided sufficient qualitative responses and meaningful quantitative data to consider the intervention acceptable. The weekly logs in the family journals were thematically analysed and four key themes were identified as having a positive impact: yoga, breathing techniques, mindfulness activities, improved connection.

**Conclusion:**

This study has highlighted promising findings relating to yoga as a family intervention.

Families reported applying the techniques, outside of the sessions to manage emotions. The family journals were a space where adults had the freedom to choose what they wrote, this method allowed us to identify the intervention had a positive impact upon family connections. Using the journals was a simple way to capture the voices of the participants.

